# Red Queen revisited: Immune gene diversity and parasite load in the asexual *Poecilia formosa* versus its sexual host species *P*. *mexicana*

**DOI:** 10.1371/journal.pone.0219000

**Published:** 2019-07-03

**Authors:** Fabian Gösser, Manfred Schartl, Francisco J. García-De León, Ralph Tollrian, Kathrin P. Lampert

**Affiliations:** 1 Department of Animal Ecology, Evolution and Biodiversity, Ruhr-University Bochum, Bochum, Germany; 2 Department of Physiological Chemistry I, Wuerzburg University, Wuerzburg, Germany; 3 Hagler Institute for Advanced Study and Department of Biology, Texas A&M University, College Station, Texas, United States of America; 4 Centro de Investigaciones Biológicas del Noroeste, S.C. (CIBNOR, S.C.), Instituto Politécnico Nacional No. 195, Col. Playa Palo de Santa Rita, La Paz, BCS, México; National Cheng Kung University, TAIWAN

## Abstract

In accordance with the Red Queen hypothesis, the lower genotypic diversity in clonally reproducing species should make them easier targets for pathogen infection, especially when closely related sexually reproducing species occur in close proximity. We analyzed two populations of clonal *P*. *formosa* and their sexual parental species *P*. *mexicana* by correlating individual parasite infection with overall and immune genotype. Our study revealed lower levels of overall genotypic diversity and marginally fewer MHC class I alleles in *P*. *formosa* individuals compared to sexually reproducing *P*. *mexicana*. Parasite load, however, differed only between field sites but not between species. We hypothesize that this might be due to slightly higher genotypic diversity in *P*. *formosa* at the innate immune system (toll like receptor 8) which is likely due to the species’ hybrid origin. In consequence, it appears that clonal individuals do not necessarily suffer a disadvantage compared to sexual individuals when fighting parasite infection.

## Introduction

Sexual reproduction is omnipresent in the majority of all animal groups [1, 2] despite the notion that it includes very high costs [3–5]. This observation led to the assumption that the costs of sexual reproduction should be outweighed by its benefits, namely genetically diverse offspring due to allele recombination and the purging of deleterious mutations (Muller’s ratchet) [3, 6]. Nevertheless the evolution and maintenance of sexual reproduction are still major questions in evolutionary biology [5, 7–9]. One major generally accepted explanation for the maintenance of sexual reproduction is the Red Queen hypothesis [1, 10]. It states that recombination results in a fitness advantage in biotic interactions. Recombination leads to fluctuating allele frequencies at loci that determine host fitness against parasite efficacy (Hamilton et al. 1990). Therefore, asexual organisms, particularly the ones co-occurring with closely related sexual species, should be an easier target for parasites and pathogens due to the lower genotypic diversity that compromises pathogen adaptation [11].

An example for an asexual species living in tight co-occurrence with a closely related species is *Poecilia formosa*. This fish originated as a hybrid of two sexual species *Poecilia latipinna* and *Poecilia mexicana* [12–14]. Even though it reproduced clonally, it still needs males of a closely related species, including the parental species, for reproduction (gynogenesis) [15, 16]. Diploid and triploid clonal lineages can be found in the field [9]. Because of its reproductive mode, *P*. *formosa* lives in tight co-occurrence and forms mixed shoals with its host species [17]. High population densities in these shoals lead to regular infection with a range of parasites and pathogens [18, 19]. A primary defense mechanism to resist pathogen infections is the immune system. Basically, pathogens or parasites are recognized, leading to signaling cascades that result in counteraction to infection [20]. The underlying immune genes play an important role in the response to pathogen infections and resistance to parasites, and consequently have been discussed as a genetic foundation on which selection can act [21].

Traditionally, innate and adaptive immunity are distinguished. The innate immune system reacts with an immediate non-specific response to pathogen infection [22, 23]. In teleost fish the innate immune system is a fundamental defense strategy and a lot of immune defense parameters are more active and have a higher diversity than in mammals [24]. The signaling-cascades of the innate immune system are triggered by “Pattern Recognition Receptors” (PRRs), which react in binding “Pathogen Associated Molecular Patterns” (PAMPs). These PAMPs include lipopolysaccharide, peptidoglycans, bacterial DNA, viral RNA and other molecules [25]. Very important PRRs are the Toll-like receptors that recognize diverse PAMPs and can activate diverse signaling-cascades to protect against pathogens and parasites (Akira et al. 2006, Lemaitre et al. 1996). The first piscine TLR gene was reported in *Carassius auratus* (Stafford et al. 2003), followed by TLR genes in *Fugu rubripes* [26] and *Danio rerio* [27]. So far 20 TLR types (TLR1, 2, 3, 4, 5M, 5S, 7, 8, 9, 13, 14, 18, 19, 20, 21, 22, 23, 24, 25, 26) have been found in a variety of teleost species [28].

In addition to the innate immune response, the adaptive immune system, can be activated. The recognition of pathogens is improved as the immune system adapts its response during an infection. The response is then memorized even after the elimination of the pathogen or parasite. This immunological memory helps the adaptive immune system to launch a more rapid and intense response the next time this same pathogen or parasite is encountered [22]. Major histocompatibility complex (MHC) genes play an important role in the recognition of pathogens [22, 29]. MHC genes have a great allelic diversity and the genotypic diversity can be extremely high at the population level. This diversity allows for the recognition of a wide range of pathogen derived protein fragments [30]. The MHC is found in all jawed vertebrates; however, the number of copies for the MHC genes can differ greatly between species [31–33]. MHC allele numbers and infection rate correlate inversely; however, some studies have shown that intermediate numbers of MHC alleles render the most effective protection against pathogen infection [34].

Immune genes are the most promising candidates to link genotypic diversity and pathogen resistance and therefore to test the Red Queen hypothesis. *P*. *formosa* provides excellent preconditions for proving or disproving the Red Queen hypothesis: sexual and clonal species occur in the same environment (mixed shoals) and are very closely related. So far, however, comparisons of clonal versus sexual *Poecilia* either focused on the analyses of parasite infections [18, 19]or the genetic diversity of immune genes [9, 35]. A simultaneous analysis of immune genotype and pathogen infection has not been done. Therefore, in this study, we investigated differences in the immune system between *P*. *formosa* and its closely related, sexual reproducing species *P*. *mexicana* and correlated those differences to parasite load in both species. As an indicator of parasite susceptibility a digenean trematode, *Uvulifer* sp., was used. All species of Poecilinae are regularly infected by this trematode, which uses water-snails as primary host, fish as a secondary host and piscivorous birds as final host [36]. Infection can be easily identified by distinct black spots on the skin of the fishes. These black spots develop because of the cercaries of *Uvulifer* sp., which penetrate the fish skin and provoke the production of a cyst around it. This event is followed by the migration of melanocytes, which lead to the appearance of the black spots. This is why infection with this parasite is also referred to as black spot disease (BSD) [36–38]. An infection with *Uvulifer* sp. is not deadly for the fishes but it is assumed that it comes with decreased energy levels for the host; also the penetration of the skin causes mechanical damage which is assumed to be costly for the host [38]. We compared the parasitic load (*Uvulifer* sp.) in *P*. *formosa* (clonal) and *P*. *mexicana* (sexual) from two different locations, the Río Purificación and the Río Guayalejo, and compared the results with the overall genotypic variability as well as the genotypic variability at two different immune gene loci: MHC class I and TLR 8. Following the Red Queen hypothesis, we expected the clonal *P*. *formosa* to have lower genotypic diversity and higher parasite loads than the sexual *P*. *mexicana*. In addition, we looked for evidence of local adaptation in the immune genes and correlations between overall and immune gene genotypes. We found that while the parasite load differed significantly between field sites, the clonal and sexual species showed similar infection rates. It seems that the hybrid origin of the clonal *P*. *formosa* conveys an immune advantage, that at the individual outweighs the disadvantages of clonal reproduction.

## Material and methods

### Ethics statement

In the field (Mexico), fish were handled very briefly to check for signs of *Uvulifer* infection and a small piece of the dorsal fin was cut for genetic analyses. All fish were released immediately after handling. The experiments complied with all laws of the country and were approved by the National Commission of Aquaculture and Fisheries (CONAPESCA) of the Mexican government (permit numbers DGOPA/16986/191205/8101 and DGOPA/02232.230706–1079).

### Origin of samples, parasite counts and DNA extraction

Samples of *Poecilia formosa* and *Poecilia mexicana* were collected at two field sites in Mexico in 2010; in the Río Purificación (P) (24°04.711'N, 99°07.410'W), and in the Río Guayalejo (G) (23°16.624'N, 98°56.315'W). Seine nets were used to capture individuals. Collected specimens were identified, black spots on the body were counted, and a small piece of the fast-regenerating dorsal fin was clipped from each individual and stored in ethanol (70%) until further analyses in the laboratory. At the Río Purificación site 190 individuals were sampled: 118 *P*. *formosa* and 72 *P*. *mexicana*. At the Río Guayalejo site 46 *P*. *formosa* and 30 *P*. *mexicana* were sampled. After parasite count and fin clip, the fishes were immediately released.

### Molecular analyses

DNA extraction was carried out using a Chelex protocol [39]. Overall genotypic diversity of the samples was determined in another (unpublished) study that included ten variable microsatellite loci (Sat1, KonD15, PR39, mATG31, mATG38, mATG44, mATG61, mATG78, mCA16 and mCA20 [40, 41]. PCR reactions contained 10 mMTris–HCl (pH 8.85), 50 mMKCl, 0.1%Triton X-100, 1.5 mMMgCl2, 0.2 mMof each dNTP, 10 pmol of each primer and 0.05 U Taq polymerase. Reactions were performed in a total volume of 10μL using the following conditions: 5 min of denaturing at 94°C, 40 cycles of 30s denaturing at 94°C 30s, 30s annealing at 52°C for KonD15, 58°C for Sat1 and 55°C for all other primers and 30s extension at 72°C, followed by a final extension of 5 min at 72°C. PCR product size was analyzed on a Licor 4300 DNA Analyzer (Licor Biosciences, NE, USA).

For the investigation of MHC class I gens and their variability within *P*. *formosa* and *P*. *mexicana* and between the two species, the exon 2 region of the MHC class I locus was examined. Exon 2 is the antigen-presenting and therefore most variable region of the MHC class I locus [42–44]. For amplification of this region primers Tu1372 (forward) and Tu1373 (reverse) were used [9, 42, 43]. The 5´ end of the forward primer Tu1372 has a GC-clamp [45], which enhances resolution and prevents a complete splitting up of the PCR product during DGGE. PCR was carried out starting with 5min at 95°C initial denaturation followed by 30 s at 95°C, 30 s at annealing temperature (45°C), 30 s 72°C for 40 cycles followed by 30 minutes final elongation to counter the presence of double bands on the DGGE-gels (see below) [46]. To test for successful amplification of the desired DNA fragments (approx. 300 bp) a horizontal gel electrophoresis was used with a 1.5% agarose gel concentration [47]. Samples where then processed using a denaturing gel gradient (DGGE) approach (DGene system, Bio-Rad). Optimal running conditions for the MHC I alleles were: 6.5% polyacrylamid solution (37.5:1 ratio acrylamid/bisacryamid), 30–60% urea gradient, run temperature 60° C, runtime 20 h and a current of 60 V. The gel was loaded with 47 μL PCR product and 8 μL loading buffer (200 μL HPLC-water, 800 μL glycerine (Roth), 0.001g bromphenole Blue-Na-salt (SERVA)). For every sample, the bands that could be observed on the gels were counted. Bands of the same height/migration distance in different samples were interpreted as having the same DNA sequence. Every band observed for an individual was counted as a gene copy and the observed band pattern for that individual was interpreted as its genotype. Samples showing the same distinct combination of bands (= banding pattern) were interpreted as having the same genotype. To adjust for small variances in running length of PCR products between different gels, the same four samples that showed a high variability of bands were chosen as a standard and run on every gel. Samples that could not be scored unambiguously were excluded from further analyses (Table 1).

**Table 1:** Number of genotypes and values for ENC, CE and CD for the diploid and triploid *P*. *formosa* and *P*. *mexicana* in the Río Guayalejo and the Río Purificación.

		Number of individuals/ Number of genotypes			ENC			CE			CD		
		MS	MHC I	TLR 8	MS	MHC I	TLR 8	MS	MHC I	TLR 8	MS	MHC I	TLR 8
**Rio Guayalejo**	***P*. *formosa* 2n**	**32/27**	**31/4**	**32/5**	**20.90**	**3.77**	**4.53**	**0.75**	**0.94**	**0.91**	**0.95**	**0.73**	**0.78**
	***P*. *formosa* 3n**	**14/7**	**13/2**	**14/1**	**3.92**	**1.90**	**1.00**	**0.56**	**0.95**	**1.00**	**0.74**	**0.47**	**0.00**
	***P*. *mexicana***	**30/30**	**26/6**	**27/2**	**30.00**	**4.76**	**1.80**	**1.00**	**0.79**	**0.90**	**0.97**	**0.79**	**0.44**
**Rio Purificacion**	***P*. *formosa* 2n**	**112/62**	**106/2**	**109/4**	**27.88**	**1.47**	**2.19**	**0.45**	**0.49**	**0.55**	**0.96**	**0.33**	**0.54**
	***P*. *formosa* 3n**	**6/2**	**6/2**	**6/1**	**1.38**	**1.38**	**1.00**	**0.69**	**0.69**	**1.00**	**0.28**	**0.28**	**0.00**
	***P*. *mexicana***	**72/69**	**66/4**	**69/2**	**66.46**	**2.47**	**1.12**	**0.96**	**0.62**	**0.56**	**0.97**	**0.59**	**0.11**

The Toll-like receptor 8 (TLR 8) shows high levels of variability in the exon 2 region. Thus primers TLR1601 5´-TGACAATGCCTTCCAGGAAC-3´ and TLR1602 5´-ACCTGCTATGTTGGACAACG-3´ that amplify this region were designed using Geneious R6 (http://www.geneious.com [48]). A GC-clamp (Sheffield et al. 1989) was attached to the 5´ end of the forward primer (TLR1601). The PCR was carried out starting with 5 min at 95°C initial denaturation followed by 30 s at 95°C, 30 s at annealing temperature (45°C), 30s 72°C for 40 cycles followed by 30 minutes final elongation. To test for successful amplification of the desired DNA fragments (ca. 500bp) a horizontal gel electrophoresis was used with a 1.5% agarose gel concentration [47]. For screening of TLR 8 diversity a urea gradient of 20–50% was used, while the other DGGE parameters were the same as described above. Four individuals were chosen as standard and run on every gel. As in the MHC analyses bands of the same height were interpreted as the same alleles and identical combinations of bands as the same genotype. Samples that could not be scored unambiguously were excluded from further analyses (Table 1). To validate our DGGE-approach and to know how many different alleles of TLR 8 could be identified, 10 samples, representing nine of the genotypes found, were sequenced, with a prior cloning step (pGem-T Easy Vector System—Promega Corp.) ensuring successful Sanger sequencing of single alleles. All sequences were edited using Geneious R6 [48]. Sequences were trimmed according to quality, and aligned using the ClustalW-algorithm [49] implemented in Geneious. The consensus sequences of all samples were then aligned and visually inspected. Obvious sequencing mistakes, gaps or inserts were corrected by hand. An allele was only considered valid if we found it at least three times in the sequencing data. The sequencing results corresponded well with the DGGE banding patterns: All unique sequences resulted in a distinguishable DGGE band and could always be scored correctly.

### Statistical analyses

For the analysis of the parasite load the two sample locations as well as both fish species were compared. First, normality distribution of the parasite load data was tested using the Shapiro-Wilk test. Since the parasite loads differed significantly from normal distribution (p-values between 1.34E-4 and 1.42E-14) we used non-parametric tests for all further analyses. Since within field sites diploid and triploid *P*. *formosa* did not vary in parasite load (Mann-Whitney U test Rio Guayalejo U = 170 p = 0,8264, Rio Purificacion U = 279 p = 0,5859) we decided to pool them. To test differences in parasite load between species within and among field sites we used a Kruskal-Wallis test followed by a Dunn’s posthoc test. Bonferroni correction was used to compensate for multiple testing.

A similar analysis was performed to test for differences in MHC allele numbers in species, ploidy and location. The Shapiro-Wilk test revealed that MHC class I allele counts were closer to normal distribution, however, still three of the six groups differed significantly from normal distribution (p-values between 2.19E-2 and 1.14E-16). A Mann-Whitney U test revealed significant differences between diploid and triploid *P*. *formosa* in the Rio Purificacion field site (Mann Whitney U = 81.5 p = 0.00026). Therefore, all six groups (diploid and triploid *P*. *formosa* and *P*. *mexicana* from each field site) were analyzed separately in the Kruskal-Wallis analyses. Dunn’s posthoc test was done to find specific differences between the groups and Bonferroni correction was used to compensate for multiple testing. All analyses were done using the program PAST version 3 [50].

Additionally, the effective number of clones (ENC), clonal diversity (CD) and clonal evenness (CE) after Menken et al. (1995) [51] were calculated for MHC class I, TLR 8, as well as for the genotypes originating from microsatellites. The ENC describes the number of clones, which actually reproduce in the population; CD describes the diversity of the population and CE the distribution of genotypes, where 1 is evenly distributed and 0 describes an uneven distribution. All analyses are based on the frequency (π) of the clonal lineages in the population: ENC = 1/(∑π^2^); CE = ENC/(Number of genotypes); CD = 1 − ∑π^2^.

In the overall genotypic diversity we expected *P*. *mexicana* to have individual genotypes and consequently CD and CE to equal 1. We expected the clonal *P*. *formosa* to show lower values. At the immune gene level we expected shared genotypes in the clonal species but potentially also in the sexual species depending on allelic diversity or potentially local selection. Furthermore, we averaged the number of MHC I alleles found for the two sampled species in the two locations to see if there was a correlation between number of MHC I alleles and parasitic load. We also investigated the distribution of immune alleles in the two field sites to potentially find evidence of local adaptation. Finally, it was tested if the genotypes found for MHC class I always occurred in combination with distinct TLR 8 genotypes. To visualize the co-occurrence of distinct MHC I genotypes with distinct TLR 8 genotypes, the R package “circlize” (Version: 0.4.3 [52]) was used.

## Results

**Fig 1:**
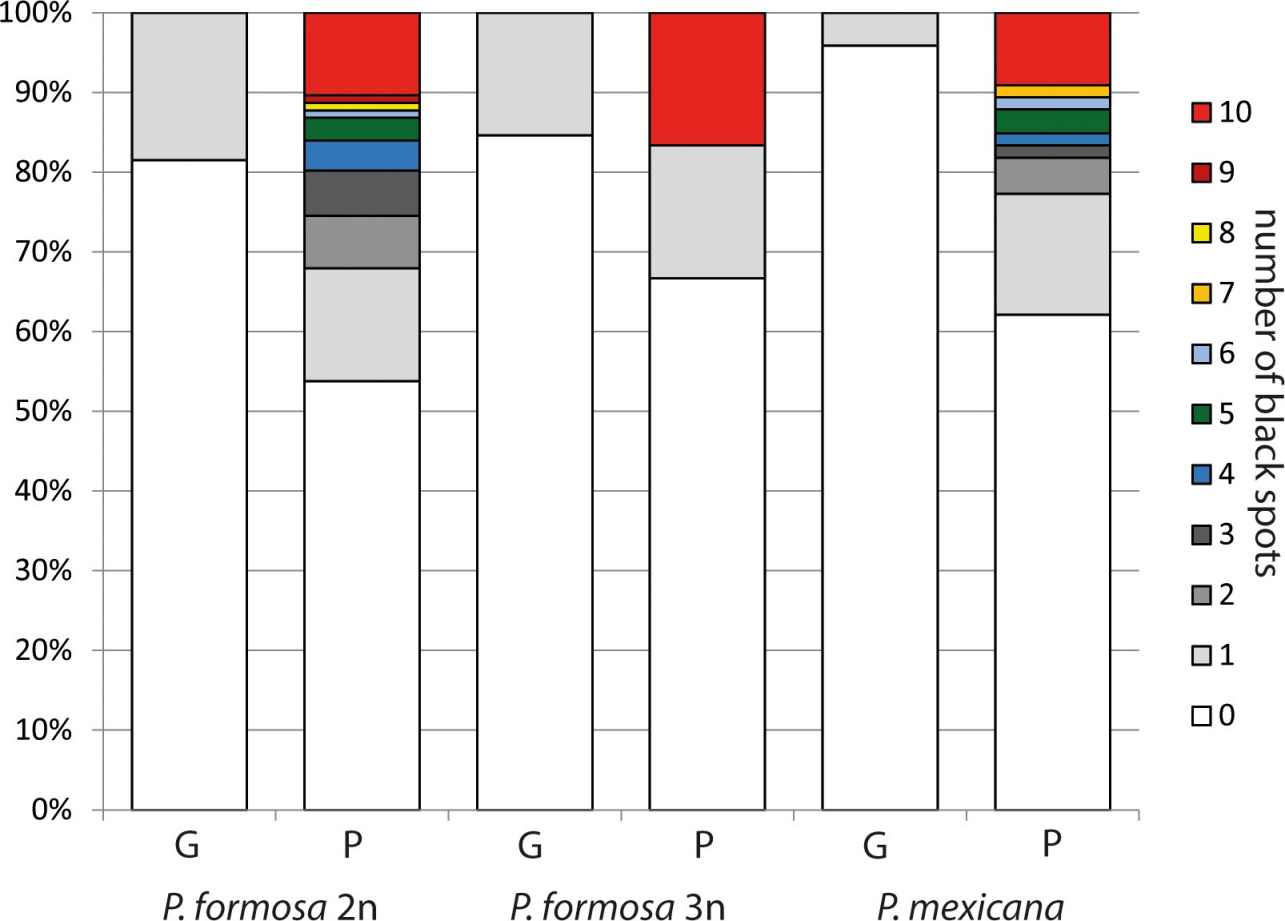
Number of parasites for diploid (2n) *P*. *formosa*, triploid *P*. *formosa* (3n) and *P*. *mexicana* of the Río Guayalejo (G) and the Río Purificación (P) field sites.

For investigation of immune gene variability within *P*. *formosa* and *P*. *mexicana* and also between the two species a total of 266 samples could be analyzed successfully. Parasitic load was higher in the Río Purificación than in the Río Guayalejo. In the Río Guayalejo field site, 78.1% of fish showed no sign of infection while in in the Río Purificación only 57.8% individuals did not show any black spots. Also in the Río Guayalejo the highest number of black spots in one individual was one, while at the Rio Purificacion site several individuals with more than 10 spots were found. While the locations clearly differed in parasite load, the species did not (Fig 1, Table 2).

**Table 2:** Parasite load. Kruskal-Wallis test for equal medians and following Dunn’s posthoc test. Dunn’s z values above the diagonal, Bonferroni corrected p-values below the diagonal. Significant p-values are marked in bold typeface.

Kruskal-Wallis test					
H(Chi^2^) = 17.38					
Hc (tie corrected) = 24.2					
p(same) = **2.272E-5**					
Dunn's posthoc test					
		Rio Guayalejo		Rio Purificacion	
Field site	Species	*P*. *formosa*	*P*. *mexicana*	*P*. *formosa*	*P*. *mexicana*
Rio Guayalejo	*P*. *formosa*		0.8355	3.643	2.481
Rio Guayalejo	*P*. *mexicana*	1		3.942	2.991
Rio Purificacion	*P*. *formosa*	**0.00162**	**0.00048**		1.120
Rio Purificacion	*P*. *mexicana*	0.07864	**0.01671**	1	

Overall genotypic diversity could be determined for all samples using the microsatellite markers. In total 144 diploid *P*. *formosa*, 20 triploid *P*. *formosa* and 102 *P*. *mexicana* from both locations were genotyped. In the Rio Guayalejo 27 unique genotypes were found for diploid *P*. *formosa* (32 individuals), while the triploids (14) showed seven different genotypes. Each of the 30 *P*. *mexicana* individuals had its own genotype (Table 1). For the Río Purificación we identified 62 genotypes for 112 diploid *P*. *formosa*, 2 different genotypes for 6 triploid *P*. *formosa* and 69 unique genotypes for *P*. *mexicana* (Table 1).Genotypic diversity was high in *P*. *mexicana* and diploid *P*. *formosa* in both field sites. Triploid *P*. *formosa* were less diverse than diploids but showed higher levels of genotypic diversity in the Rio Guayalejo field site (Table 1).

**Fig 2:**
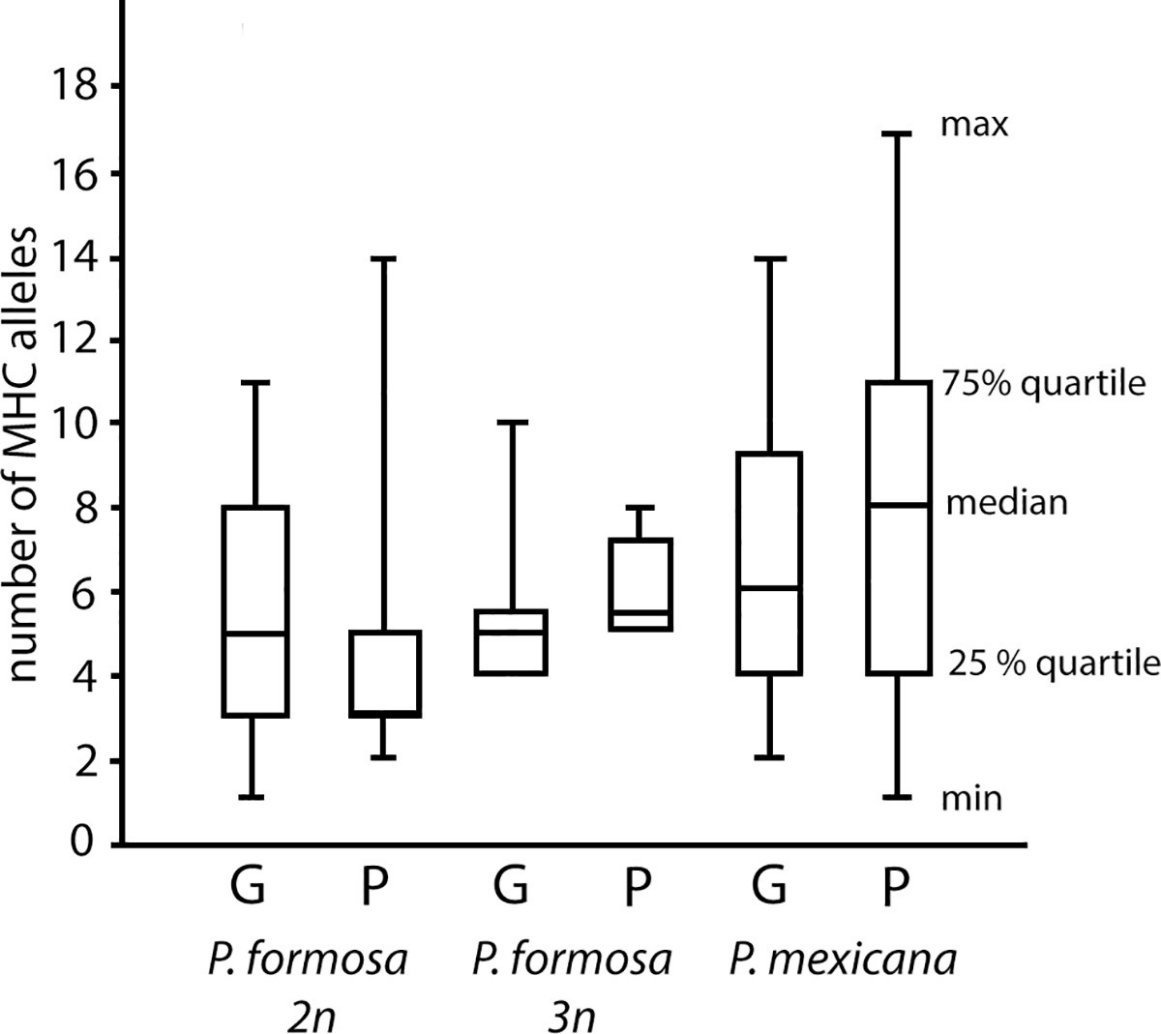
Median number of MHC I alleles for diploid (2n) and triploid (3n) *P*. *formosa* and *P*. *mexicana* for both sample locations (G—Rio Guayalejo, P–RioPurificacion). Box plots show median plus upper and lower quartile and minimum and maximum values.

**Fig 3:**
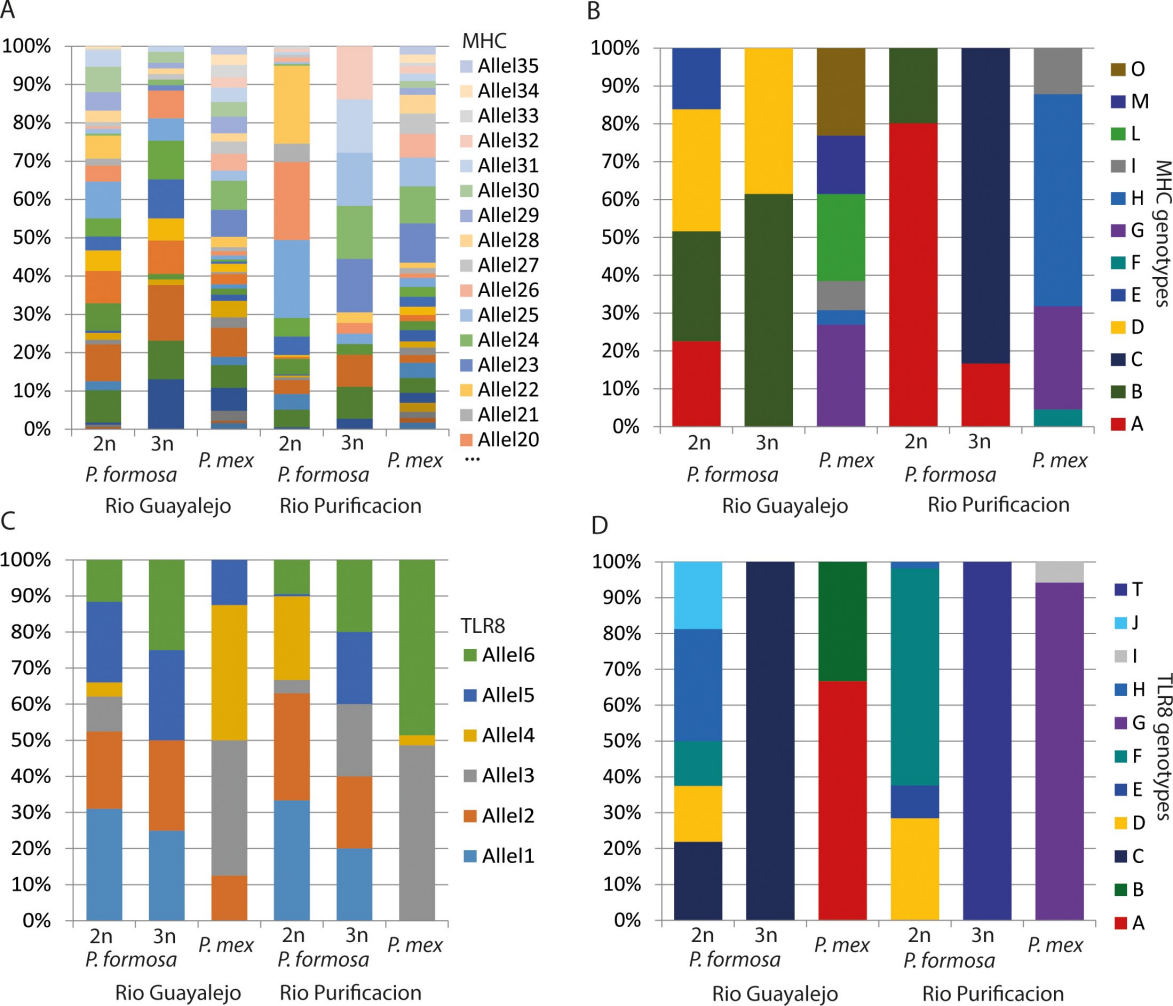
Immune gene allele and genotype frequencies in all field sites. A) MHC alleles in all locations, species and ploidy levels. B) MHC genotypes in all locations, species and ploidy levels. C) TLR 8 alleles in all locations, species and ploidy levels. D) TLR8 genotypes in all locations, species and ploidy levels. (Pmex–Poecilia mexicana, 2n –diploid, 3n –triploid).

For the MHC analysis 248 individuals yielded band patterns that allowed for further analysis. A total number of 35 MHC class I alleles were found. All alleles were present in *P*. *mexicana*, *P*. *formosa* had 31 different alleles. The highest number of different alleles for an individual was 15 in *P*. *formosa* and up to 17 different MHC alleles in *P*. *mexicana*. The triploid *P*. *formosa* had a total number of 21 different alleles with one individual having up to 10 distinguishable alleles. The median number of MHC I alleles for *P*. *formosa* was three in the Río Purificación and five in the Río Guayalejo. The triploid *P*. *formosa* had 5.5 in the Río Purificación and five in the Río Guayalejo. For *P*. *mexicana* we found the highest median number of MHC I alleles with eight in the Río Purificación and six in the Río Guayalejo (Fig 2). The Kruskal-Wallis test showed significant differences in the data set. The Dunn’s posthoc test, however, revealed that this was only due to the low number of MHC class I alleles in diploid *P*. *formosa* from the Rio Purificacion field site. There were no other significant differences between species, ploidy or location (Table 3). While *P*. *formosa* and *P*. *mexicana* had MHC I alleles in common, MHC I genotypes (distinct allele combinations) were never shared between the species (Fig 3A and 3B). We found 12 different genotypes for both species, five genotypes for *P*. *formosa* and seven genotypes for *P*. *mexicana*. Six of these genotypes were shared between the two locations, three in *P*. *formosa* and three in *P*. *mexicana*. One of the genotypes of *P*. *formosa* was only found in the Río Purificación. *P*. *mexicana* showed also one genotype exclusively in the Río Purificación and three genotypes only in the Río Guayalejo. Triploid *P*. *formosa* also had a private MHC I genotype that was not found in any of the other groups (Fig 3A and 3B). For all species and ploidy levels MHC class I diversity was higher in the Rio Guayalejo. MHC class I genotypes were also more evenly distributed in the Rio Guayalejo than in the Río Purificación (Table 1).

**Table 3:** MHC allele numbers. Kruskal-Wallis test for equal medians and following Dunn’s posthoc test. Dunn’s z values above the diagonal, Bonferroni corrected p-values below the diagonal. Significant p-values are marked in bold typeface. Rio P–Rio Purificacion, Rio G–Rio Guayalejo, P form–*Poecilia formosa*, P mex–*Poecilia mexicana*.

Kruskal-Wallis test							
H(Chi^2^) = 67.67							
Hc (tie corrected) = 65.36							
p(same) = **9.426E-13**							
Dunn's posthoc test							
		Rio G	Rio G	Rio G	Rio P	Rio P	Rio P
		*P*. *form 2n*	*P*. *form 3n*	*P*. *mex*	*P*. *form 2n*	*P*. *form 3n*	*P*. *mex*
Rio G	*P*. *form 2n*		0.4432	1.566	3.626	0.9383	2.178
Rio G	*P*. *form 3n*	1		0.7946	2.765	0.5512	1.08
Rio G	*P*. *mex*	1	1		4.946	0.004672	0.25
Rio P	*P*. *form 2n*	**0.01662**	0.08549	**1.14E-05**		2.584	7.272
Rio P	*P*. *form 3n*	1	1	1	0.1464		0.1308
Rio P	*P*. *mex*	0.441	1	1	**5.31E-12**	1	

For the analyses of innate immunity the toll like receptor 8 (TLR8) was analyzed. 257 individuals yielded bands on the DGGE-gel that could be analyzed. Individual samples showed between two and five bands, with a total of seven different bands. Eleven different genotypes could be distinguished. Seven genotypes were identified for diploid *P*. *formosa*, two genotypes were found for the triploid *P*. *formosa* and four genotypes for *P*. *mexicana*. To validate the DGGE approach ten samples from nine different genotypes were chosen for sequencing. Six different alleles could be distinguished, but more alleles are possible as certain sequences only appeared two times, therefore not reaching our threshold of three discoveries in the dataset. Two of the alleles were found only in *P*. *formosa*, while the remaining alleles were shared between the two species (Fig 3C). We found two different genotypes for *P*. *mexicana* at each location. In Rio Purificacion a large majority of individuals (65 of 69) had the same genotype, while in the Río Guayalejo the distribution was more even: 27 to 18 (Table 1). *P*. *formosa* showed five TLR 8 genotypes in Rio Guayalejo and four in Rio Purificacion. As in *P*. *mexicana* one genotype was very dominant in the Rio Purificacion, while in Rio Guayalejo genotypes were more equally distributed. Triploid *P*. *formosa* had only one genotype for both locations (= no clonal diversity) (Table 1, Fig 3D).

**Fig 4:**
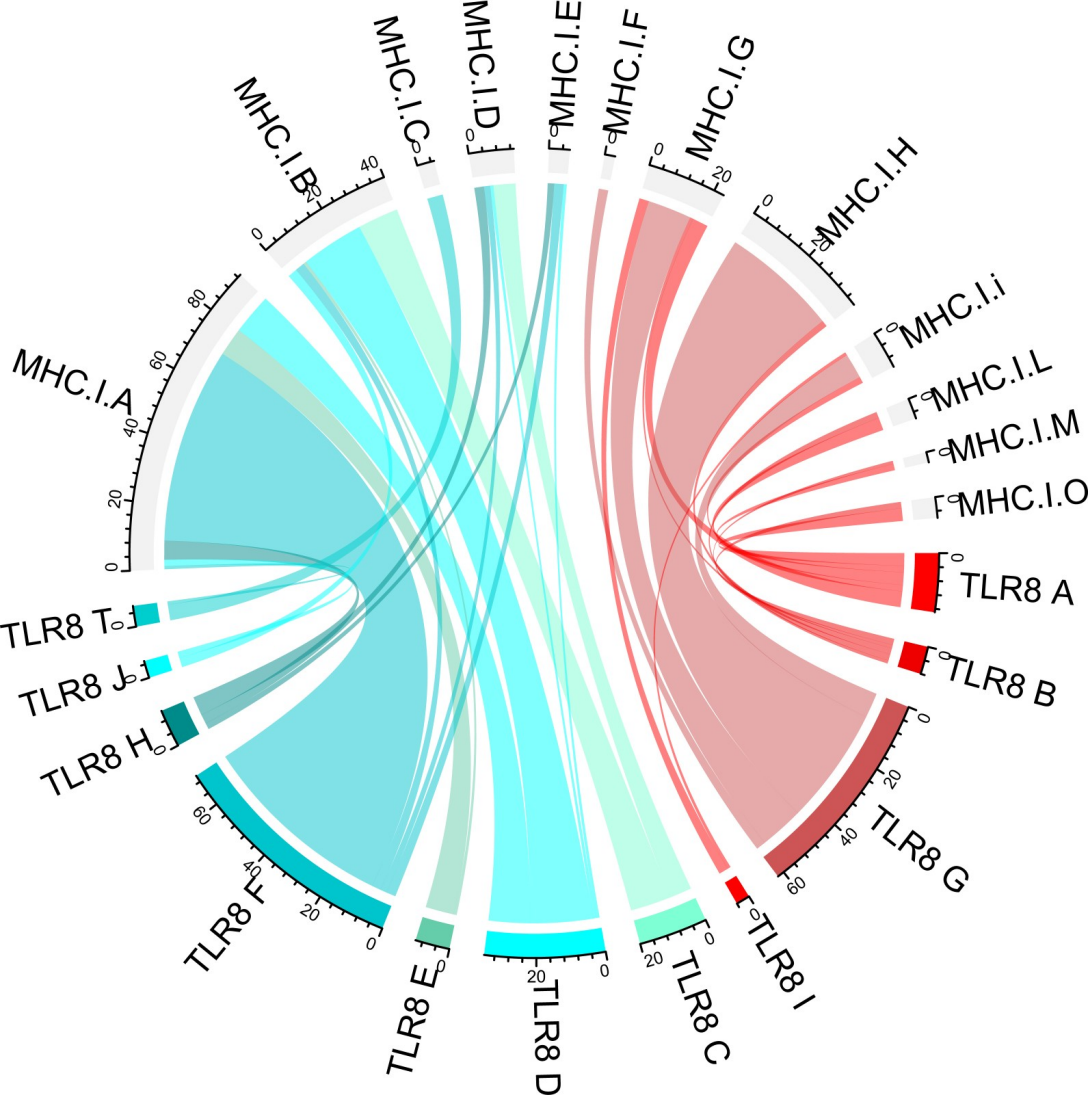
TLR8 genotypes and the corresponding MHC class I genotypes of *P*. *formosa* (blue) and *P*. *mexicana* (red). To facilitate the differentiation of alleles within species different shades of color are used.

With the genotypes found for TLR8 and MHC class I, we investigated if genotypes of the immune genes always occurred in distinct combinations. This could be indeed observed to some extent. As could be expected for a clonal organism, the diploid *P*. *formosa* that had the same genotype in the microsatellite assay also showed the same combination of MHC class I and TLR 8 genotypes. All triploid *P*. *formosa* shared the same TLR 8 genotype, but differed in their MHC class I genotype. Furthermore, we observed that TLR 8 genotypes of diploid *P*. *formosa* and *P*. *mexicana* correlated in most cases with particular MHC class I alleles with single deviations from the most common combinations (Fig 4).

## Discussion

The aim of this study was an immunogenetic analysis of the clonal fish, *Poecilia formosa* and one of its parental species, the sexually reproducing *P*. *mexicana*. In addition, parasite load (black spots—digenean trematode *Uvulifer* sp.) of both species was correlated with genotypic diversity: overall genotypic diversity (microsatellite markers) adaptive (MHC class I) and innate (TLR 8) immunity.

In contrast to our expectations based on the Red Queen hypothesis, the sexual and asexual species did not differ in parasite load. *P*. *formosa* and *P*. *mexicana* seemed to be affected by *Uvuliver* to the same degree. Interestingly, a similar observation was described earlier for the parasitic load of *P*. *formosa* in comparison to *P*. *latipinna*, the other parental species of *P*. *formosa* alongside *P*. *mexicana*. Despite looking for different macro- and micro-parasites, no significant difference could be observed between the two species [18, 19]. Studies performed on asexual geckos and closely related sexual species revealed an even lower parasitic load with mites and other parasites of the asexual geckos in comparison to the sexual species. It was proposed that asexual vertebrates may have a higher resistance to parasites because of their hybrid origin [53]. The resulting combination of genes from two different parental species could be advantageous, a phenomenon called hybrid vigor [54, 55]. This hypothesis has been has been supported by studies in hybridogenetic frogs [56] and was recently promoted for *P*. *formosa* [35].

While the clonal and the sexual species did not differ in parasite load, there was a profound difference between the locations: Both species showed a significantly higher parasitic load in the Río Purificación than in the Río Guayalejo. Grave differences in parasitic load among field sites have been reported in other studies [57]. There are two alternative explanations for this observation. First the locations could differ in parasite frequency. The differences in number of infected individuals could reflect the actual number of parasites present in the habitat. The parasite *Uvulifer* sp. has a complex life cycle and depends on the occurrence of all of its three hosts for its survival. Lemly and Esch (1984) [58] e.g. showed that the shedding of cercaria of *Uvulifer* sp. correlated with peak abundance of water snails in ponds. If one of the hosts is absent in the location or only present in low abundance the total abundance of the parasite should be impacted. As low presence of the fish host can be ruled out (personal observation on catching success [59]) the abundance of little water snails or piscivorous birds could differ greatly between the Río Purificación and the Río Guayalejo.

An alternative (second) explanation would be adaptation. Fishes in the Río Guayalejo could be better adapted to parasite infections than fishes in the Río Purificación. Several studies investigated a potential correlation of parasite load with genotypic diversity. Especially MHC genes of class I and II were in the focus so far and found evidence of local adaptation [21, 57, 60, 61].

To analyze potential genetic differences between field sites and species we analyzed the genotypic diversity of both species. For the overall genotypic diversity a microsatellite assay was used. The markers showed a high resolution in differentiating individual genotypes for *P*. *mexicana*. We are therefore confident, that *P*. *formosa* individuals with identical microsatellite genotypes belonged to the same clonal lineage. This was confirmed by the immune gene markers: *P*. *formosa* individuals with the same microsatellite genotype also shared the same immune genotype (identical MHC class I and TLR 8 combinations). Overall genotypic diversity was high in both field sites, as was immune gene diversity.

A high diversity of MHC class I alleles in fishes had been reported earlier [62]. Later, however, it was shown that that an optimum rather than a maximum number of alleles maximizes parasite resistance [21, 34]. In our study *P*. *mexicana* individuals had significantly more MHC class I alleles than *P*. *formosa*. The higher average count of MHC class I alleles in *P*. *mexicana* in comparison to *P*. *formosa* could possibly be attributed to mate-choice if females preferred males with higher MHC I allele numbers over males with lower MHC I allele numbers [21, 63]. Despite the difference in MHC allele number we did not find a species specific difference in parasite resistance: Both species were infected at similar rates. In addition, we could not find an optimal allele number correlating with low parasite susceptibility, but we noticed that within field sites a maximum number of MHC class I alleles did not maximize pathogen resistance. Interestingly, the most common MHC I genotype of *P*. *formosa*, did not show the highest parasitic load as would have been expected from the Red Queen hypothesis, where common genotypes are expected to be easier targets for parasite/pathogen infections. Instead all genotypes found for the Río Purificación showed a similar parasitic load. Especially the triploids with their very low genotypic variability should be susceptible to pathogen infections [10, 21, 40]. While other studies found that diploid *P*. *formosa* have a higher fitness than their triploid counterparts [15], it seems that the triploid fitness disadvantage is not due to higher parasite load due to common genotypes. A similar picture as for *P*. *formosa* was seen for *P*. *mexicana*. The more abundant MHC genotypes did not show enhanced parasite susceptibility as expected by the Red Queen hypothesis: All MHC genotypes (common or rare) showed similar levels of parasite infection.

In addition to MHC class I as part of the adaptive immune system, we analyzed a component of the innate immune system. We chose Toll like receptor 8 because Toll-like receptors had been shown to play a role in parasite control [64]). Eleven different genotypes were found for the TLR 8. Interestingly, the number of bands observed on the DGGE-gel were more than expected for a single copy gene: A maximum of two bands/alleles for diploids and three bands/alleles for triploids. Instead, we observed between two and five bands, with the majority of samples showing more than 2 bands/alleles. This means that in *P*. *formosa* the TLR 8 gene is most likely present in at least two copies with several alleles. Copy number variation between and even within species is quite common in teleost fishes [65, 66] and TLR 8 has been reported to exist in two different variants in zebrafish, *Danio rerio* [67, 68].

*P*. *formosa* and *P*. *mexicana* shared four of the 11 TLR 8 alleles but no genotypes, which is not surprising, considering *P*. *formosa*´s hybrid origin. The hybrid origin might also explain the slightly higher level of genotypic diversity (ENC and CD Table 1) at the TLR 8 locus in diploid *P*. *formosa* compared to sexual *P*. *mexicana*. The two alleles found only for *P*. *formosa* are likely derived from *P*. *latipinna*, the other parental species. We found two different genotypes for each location for *P*. *mexicana*. While we observed that the common genotype of *P*. *mexicana* in the Río Purificación is heavily infected and the rare genotype is infection free, which is in accordance to the Red Queen hypothesis [1], a similar pattern could not be observed for individuals in the Río Guayalejo. Also, neither the most frequent genotype of *P*. *formosa* in the Río Purificación nor Río Guayalejo showed the highest levels of parasite susceptibility.

These results are somehow unexpected from the viewpoint of the Red Queen hypothesis. It might, however, be the result of sampling time. If in the co-evolutionary dynamics of fish and parasites high frequency MHC variants have just come up, the parasites might still be lacking behind [69]. In addition, at the end of the dry season highly infected clones might already be declining and therefore might no longer be the most dominant genotypes [70, 71].

While we could not correlate certain alleles or genotypes with parasitic load or location we observed that the pattern of parasite susceptibility between the two locations matched the pattern we saw in the immune genotype diversity of MHC class I and TLR 8. We found a higher immune gene diversity but lower parasite load for both species in the Río Guayalejo than in the Río Purificación. This pattern was not reflected in the microsatellite data, even though microsatellite genotypes in *P*. *formosa* correlated with immune genotype. This finding is in accordance with the Red Queen hypothesis where we would expect high genotypic diversity to be connected with lower parasitic load.

## Conclusion

In contrast to the predictions from the Red Queen hypothesis sexually reproducing *P*. *mexicana* did not show lower levels of parasite infection than the clonally reproducing *P*. *formosa*. Instead parasite load seemed equal in both species and correlated to field site instead. Similar observations have been made before but could so far not been explained. Molecular analyses of the overall genotypic diversity showed, that while the clonal species was quite diverse it was still less diverse than the sexually reproducing species and therefore fulfilled the preconditions for the Red Queen hypothesis. More specific analyses of the immune genes of the major histocompatibility complex came to the same conclusion. Our study of a multicopy innate immune gene (TLR 8) revealed that the diploid individuals of the clonal species were even more diverse at these loci than individuals from the sexual species. This is most likely due to their hybrid origin and might balance advantages in the fight of pathogen infection.
